# F-Actin Organization and Epidermal Cell Morphogenesis in the Brown Alga *Sargassum vulgare*

**DOI:** 10.3390/ijms241713234

**Published:** 2023-08-26

**Authors:** Emmanuel Panteris, Dimitris Pappas

**Affiliations:** Department of Botany, School of Biology, Aristotle University of Thessaloniki, 54124 Thessaloniki, Greece

**Keywords:** actin filaments, brown algae, cell wall, convergence, morphogenesis, *Sargassum vulgare*, wavy epidermal cells

## Abstract

The ordinary epidermal cells of various vascular plants are characterized by wavy anticlinal wall contours. This feature has not yet been reported in multicellular algal species. Here, we found that, in the leaf-like blades of the brown alga *Sargassum vulgare*, epidermal cells exhibit prominent waviness. Initially, the small meristodermal cells exhibit straight anticlinal contour, which during their growth becomes wavy, in a pattern highly reminiscent of that found in land plants. Waviness is restricted close to the external periclinal wall, while at inner levels the anticlinal walls become thick and even. The mechanism behind this shape relies on cortical F-actin organization. Bundles of actin filaments are organized, extending under the external periclinal wall and connecting its junctions with the anticlinal walls, constituting an interconnected network. These bundles define the sites of local thickening deposition at the anticlinal/periclinal wall junctions. These thickenings are interconnected by cellulose microfibril extensions under the external periclinal wall. Apart from the wavy anticlinal contour, outward protrusions also arise on the external periclinal wall, thus the whole epidermis exhibits a quilted appearance. Apart from highlighting a new role for F-actin in cell shaping, the comparison of this morphogenetic mechanism to that of vascular plants reveals a case of evolutionary convergence among photosynthetic organisms.

## 1. Introduction

Sophisticated plant cell morphogenesis depends, among others, on cell wall properties, in particular on local thickening deposition and cellulose microfibril orientation, which establish specific axes and patterns of growth [1,2,3]. The key instrument that regulates cellulose microfibril orientation in plant cells is the cortical microtubule array [4,5,6]. Since the original discovery of cortical microtubules [7], the “alignment hypothesis” correlated cortical microtubules with cellulose microfibril orientation. The discovery of proteins interconnecting the cortical microtubules with the cellulose synthase complexes [8,9,10] has greatly enhanced the causative role of microtubules in cellulose microfibril patterning. In this context, cortical microtubules acquire specific organization configurations, allowing for the creation of a variety of particular plant cell shapes.

A prominent feature of leaf epidermis, among various vascular plant species, is the wavy anticlinal contour of ordinary epidermal cells, which has been reported in ferns, gymnosperms, dicots and monocots [11,12,13,14], in samples as early as in the Paleozoic era [15]. Although this feature exhibits variation among species [14,16], the basic morphogenetic scenario is common, relying on cortical microtubule organization and local cell wall differentiation [17,18]. After the cessation of divisions, protodermal cells, either rectangular or polygonal, grow differentially to become wavy ordinary epidermal cells, making the epidermis look like a “jigsaw-puzzle” [17,18,19]. During the above progression, cortical microtubules under the anticlinal walls are organized in bundles, while at the junctions of the external periclinal wall with the anticlinal walls they extend as radial arrays [17,18,19]. These microtubule systems define the sites where cell walls are reinforced by local thickenings, the cellulose microfibrils of which follow the orientation of the underlying microtubules [12,14,17]. Overall, this wall structure results in wavy epidermal cell morphogenesis. Meticulous and thorough studies of the above morphogenetic mechanism have revealed in detail the discrete roles of microtubules, cell wall components and several molecular regulators [14,20,21,22,23,24,25]. Moreover, a recent study expanded the presence of wavy epidermal cells in the double-layered adaxial epidermis of *Magnolia grandiflora* leaves [26]. However, no data exist about this feature in multicellular marine algae. Here we report the presence of waviness in the epidermal cells of the leaf-like blades of the brown alga *Sargassum vulgare*. To our knowledge, this is the first report of wavy epidermal cells in a photosynthetic organism not belonging to Embryophytes. 

Intriguingly, the plasma membrane of brown algal cells is not lined by cortical microtubules [27]. It was the work of Chris Katsaros’ group that first introduced F-actin as the analogue of cortical microtubules in brown algae [28,29]. While microtubules are involved in the establishment of polarity and cell division, it has been shown that cortical actin filaments should be considered responsible for cellulose microfibril orientation in several brown algal species. Accordingly, apart from analyzing the shape of epidermal cells, we investigated the organization of F-actin in growing blades of *S. vulgare*, in order to reveal any relationship between the actin filaments and wavy cell shaping. The significance and possible advantage of wavy epidermal cells in a marine multicellular alga is also discussed. 

## 2. Results

### 2.1. Epidermal Cell Wall and Shape

**Figure 1:**
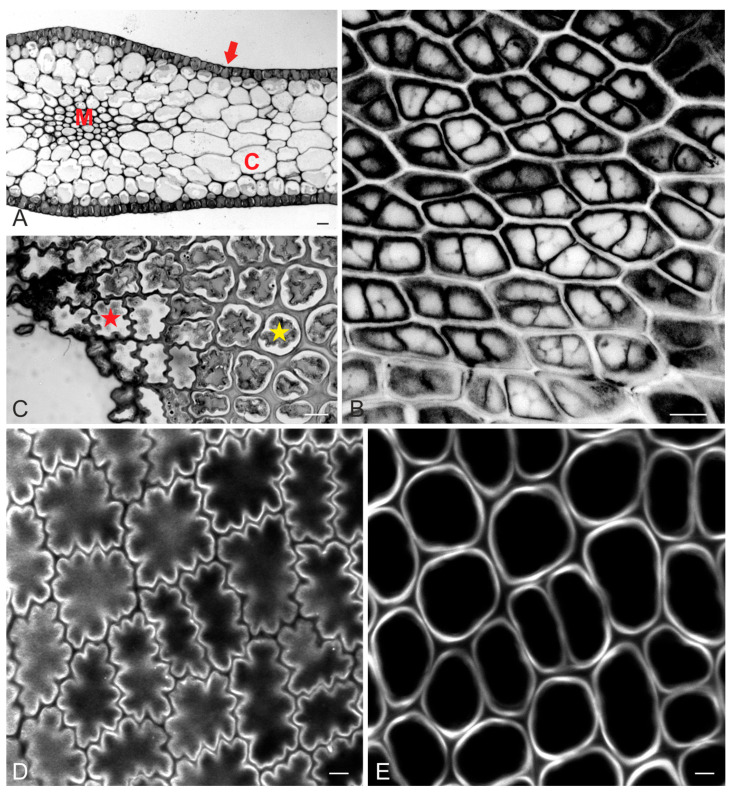
Light micrographs (**A**,**C**) and confocal laser scanning microscopy (CLSM) images after Calcofluor White staining (**B**,**D**,**E**) of young (**B**) and mature (**A**,**C**–**E**) *Sargassum vulgare* blades. (**A**) Cross-section depicting the histological pattern of the blade, consisting of epidermis (red arrow), cortex (C) and medulla (M). (**B**) Top view of meristoderm in young blade. Anticlinal walls are straight. Fluorescence intensity has been inverted for better visualization. (**C**) Tangential section of mature blade epidermis. While anticlinal walls are wavy close to the external periclinal wall (cell with red asterisk), they appear even and thick at the inner level, close to the cortex (cell with yellow asterisk). (**D**) Top-view optical section of mature blade at the junction of external periclinal and anticlinal walls. Note the prominent pattern of anticlinal wall waviness. (**E**) The same epidermal area as in (**D**), at an optical section closer to the cortex. Anticlinal walls of the same cells that are shown in (**D**) are not wavy but even and thick. Scale bars (**A**): 20 μm, (**B**,**C**): 10 μm, (**D**,**E**): 5 μm.

The blades of *Sargassum vulgare* consist of cortex and medulla cells, wrapped around by the meristoderm (or epidermis at maturity) (Figure 1A). In young blades, small meristodermal cells exhibited straight anticlinal walls at top view (Figure 1Β). After completion of growth, blade epidermal cells exhibited a prominently wavy anticlinal contour, which was restricted to the outer part, close to the external periclinal wall (Figure 1C,D). Focusing deeper towards the cortex revealed that the anticlinal walls of epidermal cells appeared gradually thicker and not wavy (Figure 1C,E). The pattern of waviness among adjacent cells was arranged so that each cell’s protrusions (lobes) coincided with its neighbor’s constrictions (necks), so that intercellular spaces did not occur between epidermal cells (Figure 1C,D). 

**Figure 2:**
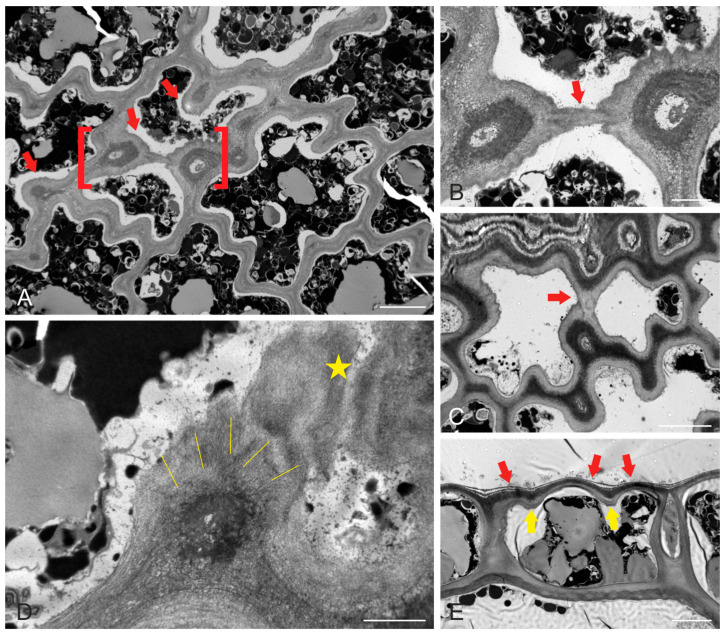
Transmission electron micrographs of mature *S. vulgare* blades. (**A**) Tangential epidermal section, depicting the deeply wavy pattern of anticlinal walls. Note the local wall thickenings (some of which are pointed to by arrows) at the constricted wall sites (“necks”). (**B**) Higher magnification of the area defined by red brackets in (**A**). Two thickenings at opposite anticlinal walls are connected through the external periclinal wall by cellulose microfibril extension (arrow). (**C**) A tangentially sectioned epidermal cell, exhibiting a similar (compare with (**A**,**B**)) connection of opposite wall thickenings (arrow) through the external periclinal wall. (**D**) Tangentially sectioned epidermal cell at high magnification, depicting a local thickening at the junction of the anticlinal wall with the external periclinal one. Cellulose microfibrils in the thickening exhibit a radial orientation (labeled by lines). The asterisk notes a cellulose microfibril extension through the external periclinal wall, connecting with a neighboring thickening (in the right half of the micrograph). (**E**) Cross-section of an epidermal cell close to the junction of the external periclinal and anticlinal walls. Local thickening profiles can be observed (yellow arrows). The external periclinal wall exhibits protrusions (red arrows) in the areas between the thickenings. Scale bars (**A**): 5 μm, (**B**): 2 μm, (**C**): 5 μm, (**D**): 1 μm, (**E**): 5 μm.

**Figure 3:**
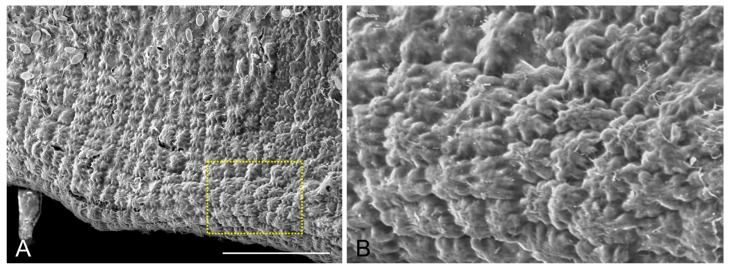
Scanning electron micrograph of mature *S. vulgare* blade, depicting the “quilted” appearance of the epidermis. In (**B**) the area defined by the rectangle in (**A**) is shown at a higher magnification. Scale bar 100 μm.

Prominent thickenings of the cell walls were present at the sites of the necks, at the junctions of anticlinal and periclinal walls (Figure 2A). These thickenings contained radially oriented cellulose microfibrils, which extended and interconnected the thickenings in a unified network (Figure 2B–D). As a result of this wall reinforcement, the external periclinal wall also protruded outwards, between the thickened areas (Figure 2E). The overall outcome of this morphogenetic process was a “quilted” pattern, exhibited by epidermal cells on their outer surface (Figure 3).

### 2.2. F-Actin Organization and Epidermal Cell Morphogenesis

**Figure 4:**
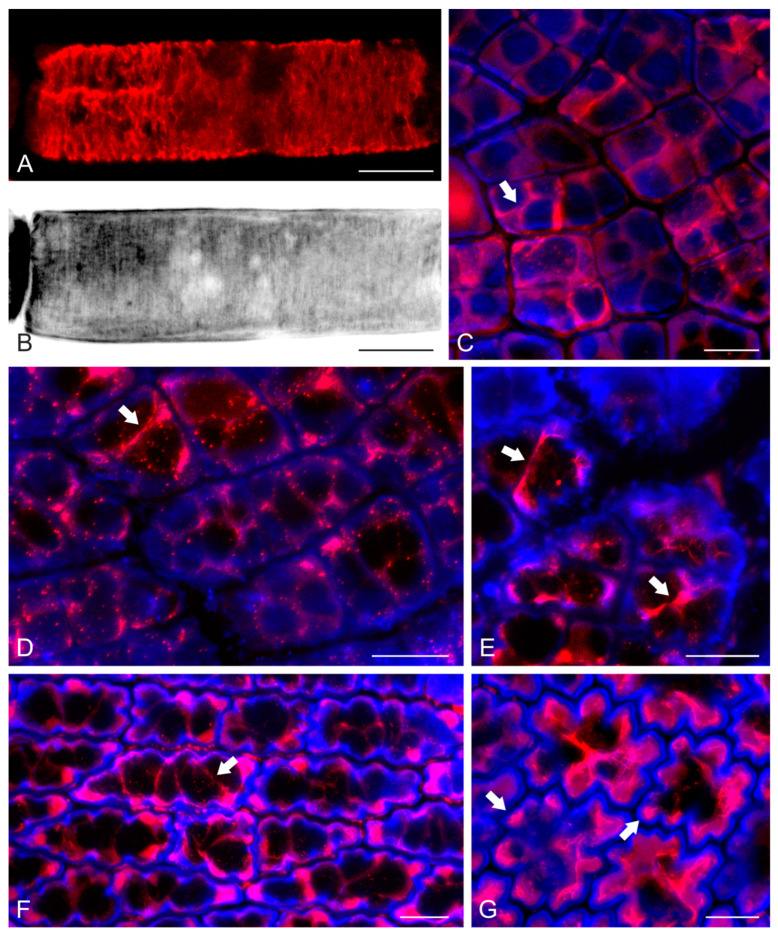
CLSM images of *S. vulgare* blades after F-actin staining with phalloidin (red) and cell wall staining with Calcofluor White (blue). In (**C**–**G**) top blade view is depicted. (**A**,**B**), Paraphysis cell. In (**B**) the wall staining is depicted in grayscale, inverted in negative to reveal the orientation of cellulose microfibrils. Both F-actin (**A**) and cellulose microfibrils (**B**) are oriented perpendicularly to the cell axis. (**C**,**D**) Meristodermal areas depicting young cells without any waviness at their anticlinal contour yet. F-actin bundles (some of which are pointed to by arrows) interconnect the anticlinal walls under the external periclinal one. (**E**) Growing meristodermal cells with slightly wavy anticlinal walls. F-actin bundles (arrows) persist. (**F**,**G**) Fairly wavy epidermal cells of mature blades. Extensive network of F-actin bundles (arrow in (**F**)) interconnect opposite wall thickenings at cell “necks”. In addition, F-actin aggregations (arrows in (**G**)) line the concave wall area of protruding cell lobes. Scale bars 10 μm.

While in higher plants cellulose microfibril orientation is regulated by cortical microtubules (see Introduction), in brown algae cellulose microfibril patterning has been correlated with F-actin organization [27,28,29]. According to our observations, in elongating cells of *S. vulgare*, like those of paraphyses, cortical actin filaments were found to be oriented perpendicularly to the cell axis (Figure 4A). In the same cells, cellulose microfibrils in the cell wall followed the same perpendicular orientation responsible for the elongated cell shape (Figure 4B). In young meristodermal cells still exhibiting straight walls, cortical F-actin was organized in bundles at the junctions of anticlinal walls with the external periclinal one, interconnected under the latter wall as a unified network (Figure 4C,D). During the growth of the meristodermal cells, their shape became wavy. In such cells, cortical F-actin bundles appeared to line the wall thickenings at the necks of the anticlinal walls of each cell, also interconnecting these thickenings by extending under the external periclinal wall (Figure 4E,F). The F-actin bundles persisted in mature epidermal cells (Figure 4G), in which F-actin aggregations were also observed in the tips of anticlinal wall lobes (Figure 4G).

## 3. Discussion

In higher plants, cell morphogenesis is orchestrated by the organization of cortical microtubules, which regulate local cell wall thickening and cellulose microfibril orientation (See Introduction). As a sculptor that creates a statue from within, the confined protoplast dictates a molding pattern on the surrounding cell wall, bringing about the ultimate cell shape. Among the most studied of such “statues” are ordinary leaf epidermal cells, which exhibit a wavy anticlinal contour, occurring through coordinating cortical microtubules, cell wall differentiation and mechanical parameters [20,21,22,23,24,30]. 

**Figure 5:**
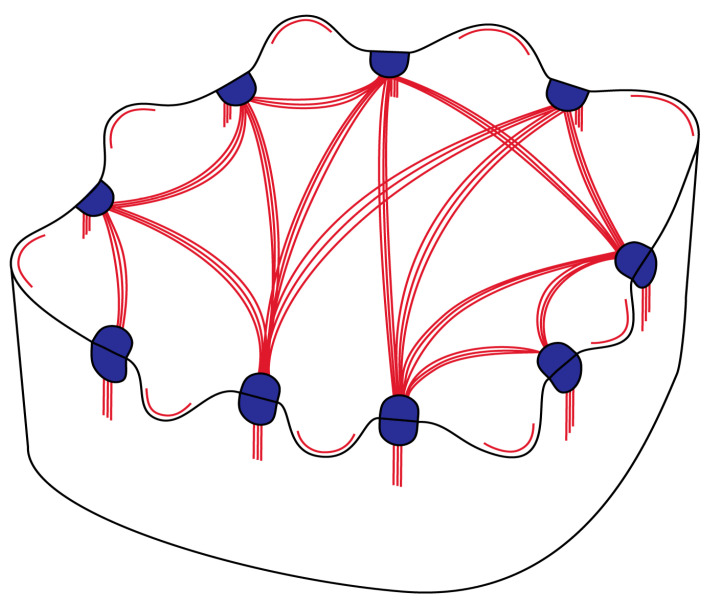
Drawing presenting an epidermal cell of *S. vulgare* blade in 3-D. F-actin bundles are shown in red and F-actin aggregations lining the concave wall of protruding lobes are shown as red lines. The local wall thickenings at the junctions of anticlinal and external periclinal walls are shown in blue. Note that waviness is restricted close to the external (upper) cell surface.

As a major difference to higher plants, brown algal cells do not host cortical microtubules under the plasma membrane. Instead, cortical actin filaments have been found to play the relevant role [27,28,29]. Here, apart from confirming these original findings by observations in elongated paraphysis cells, we report a far more complicated F-actin patterning, resulting in a particular epidermal cell shaping (Figure 5), reminiscent of that of land plants. Apart from the obvious difference in the cytoskeletal component that regulates local cell wall reinforcement (F-actin vs. microtubules), some fine details of the morphogenetic patterning can be also underlined. 

At first, the fact that anticlinal wall waviness is restricted very close to the external periclinal wall is not unique or unexpected. This wall patterning is usual among monocots, prominently present, among others, in leaves of *Cyperus* [14]. However, in the blades of *S. vulgare* this feature is also accompanied by protrusions of the external periclinal wall outwards. Although this external anaglyph could be attributed to the unique characteristics of brown algal cell walls (multi-layered construction, absence of cuticle, contents other than pectins and hemicelluloses), it is obvious that the development of lobes and constrictions does not prevent outward bulging, as suggested for plant leaf epidermal cells [31].

While in leaf epidermal cells of plants the most “effective” cytoskeletal arrays that drive wavy morphogenesis are radial microtubule clusters, at the junctions of periclinal/anticlinal walls [17,19], such a radial arrangement was not observed in the F-actin organization presented here. However, it could be suggested that the laterally interconnected F-actin bundle extensions (Figure 5), initiated at the junctions of anticlinal/periclinal walls, may provide the scaffold for radial cellulose microfibril deposition in the wall thickenings. Furthermore, the F-actin bundle extensions at the external periclinal cell surface regulate the deposition of cellulose microfibril bands in the external periclinal wall areas just over the actin filaments. These bands interconnect the local thickenings at the anticlinal/periclinal wall junctions, forming a unified cell wall reinforcement framework that resembles the “boning of a corset”. Consequently, growth is prevented along microfibril bands, while it is allowed in the wall area between them, resulting in anticlinal waviness and periclinal protrusions. 

Although in the epidermal cells of plant leaves microtubules are the major cytoskeletal component for morphogenesis, F-actin also seems to play a role during late developmental stages. It has been reported that during lobe protrusion, “patches” of actin filaments internally line the concave area of the lobe tips [17,18]. It has been suggested that these patches, also present in protruding subsidiary cells of the stomatal complexes in grasses, probably offer some kind of structural protection to the extending plasma membrane [32]. Similarly, F-actin aggregations were also observed here at advanced stages of lobe tip protrusion in blade epidermal cells of *S. vulgare*. It can therefore be hypothesized that the conditions at the lobe tips are similar among the two types of growing epidermal cells, thus requiring a similar level of plasma membrane protection. 

Taken together, apart from the differences between the morphogenetic mechanisms, wavy anticlinal wall contour appears to be a common trait in epidermal cells of plant leaves and epidermal cells of *S. vulgare* blades. It has been suggested that this feature might be advantageous for plant leaves, in terms of mechanical rigidity: wavy leaf epidermal cells may expand like “springs”, reversibly changing their shape [33], thus protecting epidermal integrity against environmental challenges, such as stretching due to wind [34,35]. In a marine environment, waves and currents exert forces similar to those caused by wind in terrestrial ecosystems. Accordingly, the blade epidermal cell waviness could be considered an advantageous feature, increasing blade rigidity and epidermal cell flexibility. In terms of evolution, the wavy anticlinal epidermal contour in this brown alga, a phenotype similar to that found in several plant species, can be considered as a case of convergence. 

## 4. Materials and Methods

### 4.1. Sample Collection–Chemicals and Reagents

Samples of *Sargassum vulgare* were collected from a rocky coastal site at Epanomi’s port (40.406182, 22.893795), Thermaikos Gulf, Greece. The samples were transferred to the lab in plastic containers full of sea water and were kept for 2 to 3 days after collection with aeration. Some blades were immediately prepared for light microscopy, transmission electron microscopy (TEM) and scanning electron microscopy (SEM), as well as for examination with CLSM. Preparation for CLSM was also repeated in blades kept in the lab. Collection was repeated 5 times, while preparation included blades of at least 10 samples each time. The chemicals and reagents used in sample preparation were purchased from Applichem (Darmstadt, Germany) and Sigma (St. Louis, MO, USA), unless otherwise stated. All preparation steps were performed at room temperature, unless otherwise specified.

### 4.2. Light Microscopy and TEM

For light microscopy and TEM, young and mature blades were cut into pieces ~3 × 3 mm^2^ and fixed immediately with 3% (*v*/*v*) glutaraldehyde (Polysciences, Warrington, PA, USA) in 50 mM sodium cacodylate buffer + 4% NaCl, pH 7, for 4 h. After 3 × 15 min rinses in the same buffer, the blade pieces were post fixed at 4 °C overnight with 1% (*w*/*v*) osmium tetroxide in the same buffer. After 3 × 15 min rinses in the same buffer, the blade pieces were dehydrated in an acetone series, treated twice with propylenoxide and finally embedded in Spurr’s resin. Semithin sections (1.5–2 μm thick) were cut with glass knives, stained with 1% (*w*/*v*) toluidine blue O in 1% (*w*/*v*) borax solution and observed under a Zeiss Axio.Imager Z2 (Zeiss, Oberkochen, Germany) light microscope. Digital micrographs were recorded with a Zeiss Axiocam MRc5 (Zeiss, Oberkochen, Germany), with AxioVision version 4.8 software, following the instructions of the manufacturer.

Ultrathin (60–90 nm) sections were cut with a diamond knife, collected on copper grids, double stained with 2% (*w*/*v*) uranyl acetate in 70% (*v*/*v*) ethanol solution and 1% (*w*/*v*) Reynolds’ lead citrate, and examined with a JEOL JEM 1011 TEM (JEOL, Tokyo, Japan) at 80 kV. TEM micrographs were recorded with a GATAN 500 digital camera (Gatan, Pleasanton, CA, USA), using the Digital Micrograph 3.11.2 software, according to the manufacturer’s instructions.

### 4.3. Scanning Electron Microscopy (SEM)

For SEM, mature blades were cut into small (~2 × 2 mm^2^) pieces, immediately fixed for 1 h in 4% (*w*/*v*) paraformaldehyde in PBS and then washed thrice (3 × 15 min) with the same buffer. For better imaging, fixed specimens were gently brushed and rinsed with distilled water to remove excess epiphytes, then wiped carefully with filter paper. Cleared samples were frozen in liquid nitrogen and directly observed under a Hitachi TM4000Plus II SEM (Hitachi, Tokyo, Japan) at 10 kV. Micrographs were acquired with the TM4000 software.

### 4.4. F-Actin and Cell Wall Fluorescent Labelling—Confocal Microscopy

For the fluorescent staining of F-actin, the blades were cut into small (~2 × 2 mm^2^) pieces, which were immediately treated for 20 min in the dark with 300 μM m-maleimidobenzoyl-N-hydroxysuccinimide ester in PEMS buffer (50 mM PIPES, 5 mM EGTA, 5 mM MgSO_4_, 4% NaCl, pH 6.8) + 0.1% Triton X-100 (*v*/*v*) to stabilize actin filaments. Afterwards, the samples were fixed for 1 h in 4% (*w/v*) paraformaldehyde + 5% (*v*/*v*) dimethylsulfoxide (DMSO) + 0.1% (*v*/*v*) Triton X-100. DyLight 554-phalloidin (Cell Signaling Technology, Danvers, MA, USA) at 1:400 was added to the fixative for better preservation of F-actin. After 3 × 15 min rinses with PEMS, the samples were extracted with 5% (*v*/*v*) DMSO + 1% (*v*/*v*) Triton X-100 in PBS for 1 h and afterwards F-actin was stained with DyLight 554-phalloidin 1:40 in PBS + 0.1% (*v*/*v*) Triton X-100 for 2 h at 37 °C in the dark. The cell wall was then counterstained with 0.05% (*w*/*v*) Calcofluor White in PBS for 10 min. After a final rinse, the samples were mounted on microscope slides in a drop of antifade solution (glycerol + PBS (2:1, *v*/*v*) + 0.5% (*w*/*v*) p-phenylenediamine). The above fluorescent specimens were examined using a Zeiss Observer.Z1 inverted microscope, equipped with a LSM780 CLSM module and ZEN2011 black edition software. Digital micrographs were recorded following the instructions of the manufacturer.

## 5. Conclusions

According to the observations of this work, epidermal cell waviness is a feature not only of vascular plants, but also found in at least one species of multicellular algae: *Sargassum vulgare*. While the morphogenetic mechanism of vascular plants involves specialized cortical microtubule organization, in brown algal cells F-actin undertakes this role. The similarity of cell shaping, though achieved by a different mechanism, reveals a case of evolutionary convergence between brown algae and vascular plants: it can be hypothesized that the wavy epidermal cell shape is advantageous against mechanical stresses, whether imposed in the marine or in the terrestrial environment. It is thus a challenge to investigate further cases of multicellular marine algae with leafy structures, to elucidate the expansion and possible deviations of wavy epidermal cell occurrence among photosynthetic organisms.

## Data Availability

Not applicable.
